# Evaluating Identity Disclosure Risk in Fully Synthetic Health Data: Model Development and Validation

**DOI:** 10.2196/23139

**Published:** 2020-11-16

**Authors:** Khaled El Emam, Lucy Mosquera, Jason Bass

**Affiliations:** 1 School of Epidemiology and Public Health Faculty of Medicine University of Ottawa Ottawa, ON Canada; 2 Children's Hospital of Eastern Ontario Research Institute Ottawa, ON Canada; 3 Replica Analytics Ltd Ottawa, ON Canada

**Keywords:** synthetic data, privacy, data sharing, data access, de-identification, open data

## Abstract

**Background:**

There has been growing interest in data synthesis for enabling the sharing of data for secondary analysis; however, there is a need for a comprehensive privacy risk model for fully synthetic data: If the generative models have been overfit, then it is possible to identify individuals from synthetic data and learn something new about them.

**Objective:**

The purpose of this study is to develop and apply a methodology for evaluating the identity disclosure risks of fully synthetic data.

**Methods:**

A full risk model is presented, which evaluates both identity disclosure and the ability of an adversary to learn something new if there is a match between a synthetic record and a real person. We term this “meaningful identity disclosure risk.” The model is applied on samples from the Washington State Hospital discharge database (2007) and the Canadian COVID-19 cases database. Both of these datasets were synthesized using a sequential decision tree process commonly used to synthesize health and social science data.

**Results:**

The meaningful identity disclosure risk for both of these synthesized samples was below the commonly used 0.09 risk threshold (0.0198 and 0.0086, respectively), and 4 times and 5 times lower than the risk values for the original datasets, respectively.

**Conclusions:**

We have presented a comprehensive identity disclosure risk model for fully synthetic data. The results for this synthesis method on 2 datasets demonstrate that synthesis can reduce meaningful identity disclosure risks considerably. The risk model can be applied in the future to evaluate the privacy of fully synthetic data.

## Introduction

### Data Access Challenges

Access to data for building and testing artificial intelligence and machine learning (AIML) models has been problematic in practice and presents a challenge for the adoption of AIML [1,2]. A recent analysis concluded that data access issues are ranked in the top 3 challenges faced by organizations when implementing AI [3].

A key obstacle to data access has been analyst concerns about privacy and meeting growing privacy obligations. For example, a recent survey by O’Reilly [4] highlighted the privacy concerns of organizations adopting machine learning models, with more than half of those experienced with AIML checking for privacy issues. Specific to health care data, a National Academy of Medicine/Government Accountability Office report highlights privacy as presenting a data access barrier for the application of AI in health care [5].

Anonymization is one approach for addressing privacy concerns when making data available for secondary purposes such as AIML [6]. However, there have been repeated claims of successful re-identification attacks on anonymized data [7-13], eroding public and regulator trust in this approach [13-22].

Synthetic data generation is another approach for addressing privacy concerns that has been gaining interest recently [23,24]. Different generative models have been proposed, such as decision tree–based approaches [25] and deep learning methods like Variational Auto Encoders [26,27] and Generative Adversarial Networks (GANs) [28-31].

There are different types of privacy risks. One of them is identity disclosure [23,24,32], which in our context means the risk of correctly mapping a synthetic record to a real person. Current identity disclosure assessment models for synthetic data have been limited in that they were formulated under the assumption of partially synthetic data [33-39]. Partially synthetic data permit the direct matching of synthetic records with real people because there is a one-to-one mapping between real individuals and the partially synthetic records. However, that assumption cannot be made with *fully* synthetic data whereby there is no direct mapping between a synthetic record and a real individual.

Some researchers have argued that fully synthetic data does not have an identity disclosure risk [29,40-46]. However, if the synthesizer is overfit to the original data, then a synthetic record can be mapped to a real person [47]. Since there are degrees of overfitting, even a partial mapping may represent unacceptable privacy risk. Therefore, identity disclosure is still relevant for fully synthetic data.

Another type of privacy risk is attribution risk [42,47], which is defined as an adversary learning that a specific individual has a certain characteristic. In this paper, we present a comprehensive privacy model that combines identity disclosure and attribution risk for fully synthetic data, where attribution is conditional on identity disclosure. This definition of privacy risk is complementary to the notion of membership disclosure as it has been operationalized in the data synthesis literature, where similarity between real and synthetic records is assessed [28,48]. We then demonstrate the model on health data.

### Background

Key definitions and requirements will be presented, followed by a model for assessing identity disclosure risk. As a general rule, we have erred on the conservative side when presented with multiple design or parameter options to ensure that patient privacy would be less likely to be compromised.

#### Definitions—Basic Concepts

**Figure 1:**
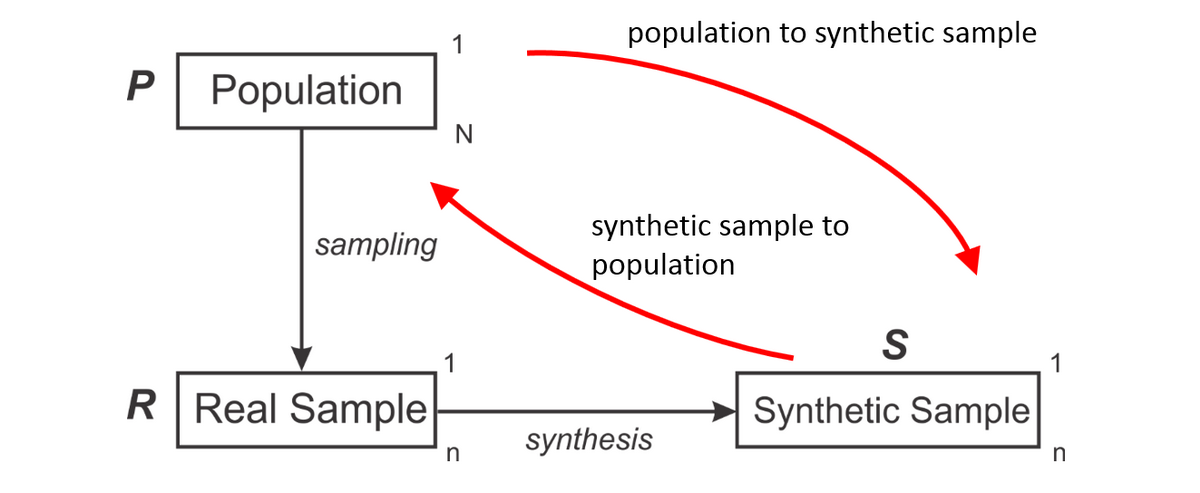
The relationships between the different datasets under consideration. Matching between a synthetic sample record and someone in the population goes through the real sample and can occur in 2 directions.

The basic scheme that we are assuming is illustrated in Figure 1. We have a real population denoted by the set *P* of size *N*. A real sample *R* exists such that *R*⊆*P*, and that is the set that we wish to create a synthetic dataset *S* from. Without loss of generality, the real and synthetic samples are assumed to be the same size, *n*.

The data custodian makes the synthetic sample available for secondary purposes but does not share the generative model that is used to produce the synthetic sample. Therefore, our risk scenario is when the adversary only has access to the synthetic data.

Synthetic records can be identified by matching them with individuals in the population. When matching is performed to identify synthetic records, that matching is done on the *quasi-identifiers*, which are a subset of the variables and are known by an adversary [49]. For example, typically, a date of birth is a quasi-identifier because it is information about individuals that is known or that is relatively easy for an adversary to find out (eg, from voter registration lists [50]). More generally, an adversary may know the quasi-identifiers about an individual because that individual is an acquaintance of the adversary or because the adversary has access to a population database or registry of identifiable information.

The variables that are not quasi-identifiers will be referred to as *sensitive variables*. For example, if a dataset has information about drug use, that would be a sensitive variable that could cause harm if it was known. In general, we assume that sensitive values would cause some degree of harm if they become known to an adversary.

To illustrate the privacy risks with fully synthetic data, consider the population data in Table 1. Individuals in the population are identifiable through their national IDs. We will treat the variable of one’s origin as a quasi-identifier and one’s income as the sensitive value. Table 2 displays the records from the real sample, and Table 3 presents records for the synthetic sample.

As can be seen, there is only one North African individual and one European individual in the population, and they both are in the real sample. Therefore, these unique real sample records would match 1:1 with the population and, therefore, would have a very high risk of being identified. The population-unique European and North African records are also in the synthetic data, and thus, here we have a 1:1 match between the synthetic records and the population.

The sensitive income value in the synthetic sample is very similar to the value in the real sample for the North African record. Therefore, arguably, we also learn something new about that individual. The sensitive income value is not so close for the European record, and therefore, even though we are able to match on the quasi-identifier, we will not learn meaningful information about that specific individual from synthetic data.

**Table 1:** Example of a population dataset, with one’s origin as the quasi-identifier and one’s income as the sensitive variable.

National ID	Origin	Income ($)
1	Japanese	110k
2	Japanese	100k
3	Japanese	105k
4	North African	95k
5	European	70k
6	Hispanic	100k
7	Hispanic	130k
8	Hispanic	65k

**Table 2:** Example of a real sample, with one’s origin as the quasi-identifier and one’s income as the sensitive variable.

Origin	Income ($)
European	70k
Japanese	100k
Hispanic	130k
Hispanic	65k
North African	95k

**Table 3:** Example of a synthetic sample, with one’s origin as the quasi-identifier and one’s income as the sensitive variable.

Origin	Income ($)
Japanese	115k
Japanese	120k
North African	100k
European	110k
Hispanic	65k

This example illustrates that it is plausible to match synthetic sample records with individuals in the population and thus identify these individuals, since a synthesized record can have the same value as a real record on quasi-identifiers. However, such identification is only meaningful if we learn somewhat correct sensitive information about these matched individuals. Learning something new is considered when evaluating identifiability risks in practical settings [51] and is part of the definition of identity disclosure [52]. Learning something new is also similar to the concept of attribution risk as it has been operationalized in the data synthesis literature [42,47].

### Counting Matches

To formulate our model, we first need to match a synthetic sample record with a real sample record. Consider the synthetic sample in Table 3 with a single quasi-identifier, one’s origin; we want to match the record with the “Hispanic” value with the real sample in Table 2. We find that there are 3 matching records in the real sample. Without any further information, we would select one of the real sample records at random, and therefore, the probability of selecting any of the records is one-third. However, there is no correct selection here. For example, we cannot say that the third record in the real sample is the correct record match, and therefore the probability of a correct match is one-third; there is no 1:1 mapping between the fully synthetic sample records and the real sample records.

The key information here is that there was a match—it is a binary indicator. If there is a match between real sample record *s* and a synthetic sample record, we can use the indicator *I_s_* (which takes on a value of 1 if there is at least one match, and 0 otherwise).

### Direction of Match

A concept that is well understood in the disclosure control literature is that the probability of a successful match between someone in the population and a real record will depend on the direction of the match [53]. A randomly selected person from the real sample will always have an equivalent record in the population. However, a randomly selected record in the population may not match someone in the real sample due to sampling. The former is referred to as a sample-to-population match, and the latter as a population-to-sample match.

In our hypothetical example, an adversary may know Hans in the population and can match that with the European record in the synthetic sample through the real sample. Or the adversary may select the European record in the synthetic sample and match that with the only European in a population registry through the real sample, which happens to be Hans. Both directions of attack are plausible and will depend on whether the adversary already knows Hans as an acquaintance or not.

Now we can combine the 2 types of matching to get an overall match rate between the synthetic record and the population: the synthetic sample–to–real sample match and the real sample–to–population match, and in the other direction. We will formalize this further below.

### Measuring Identification Risk

We start off by assessing the probability that a record in the real sample can be identified by matching it with an individual in the population by an adversary. The population-to-sample attack is denoted by *A* and the sample-to-population attack by *B*.

Under the assumption that an adversary will only attempt one of them, but without knowing which one, the overall probability of one of these attacks being successful is given by the maximum of both [49]:

**max(*A,B*)** (1)

The match rate for population-to-sample attacks is given by El Emam [49] (using the notation in Table 4):

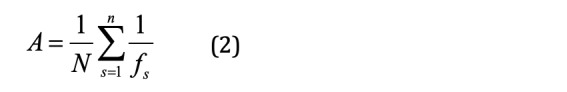



This models an adversary who selects a random individual from the population and matches them with records in the real sample. A selected individual from the population may not be in the real sample, and therefore, the sampling does have a protective effect.

Under the sample-to-population attack, the adversary randomly selects a record from the real sample and matches it to individuals in the population. The match rate is given by El Emam [49]:

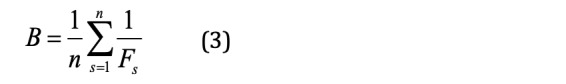



We now extend this by accounting for the matches between the records in the synthetic sample and the records in the real sample. Only those records in the real sample that match with a record in the synthetic sample can then be matched with the population. We define an indicator variable, *I_s_*=1, if a real sample record matches a synthetic sample record. Therefore, we effectively reduce the real sample to those records which match with at least 1 record in the synthetic sample. The population-to-synthetic sample identification risk can thus be expressed as

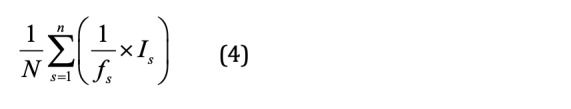



And similarly, the synthetic sample-to-population identification risk can be expressed as

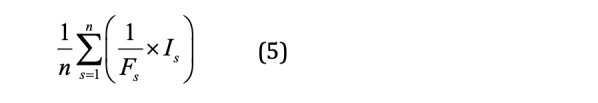



And then we have the overall identification risk from equation (1):

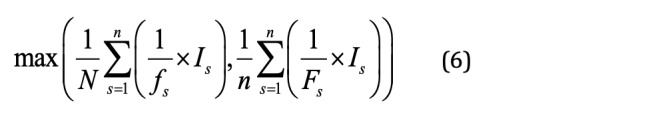



The population value of 1/*F* can be estimated using methods described in various disclosure control texts [49,54-59].

**Table 4:** Notation used in this paper.

Notation	Interpretation
*s*	An index to count records in the real sample
*t*	An index to count records in the synthetic sample
*N*	The number of records in the true population
*f_s_*	The equivalence class group size in the real sample for a particular record *s* in the real sample. The equivalence class is defined as the set of records with the same values on the quasi-identifiers.
*F_s_*	The equivalence group size in the population that has the same quasi-identifier values as record *s* in the real sample. The equivalence class is defined as the set of records with the same values on the quasi-identifiers.
*n*	The number of records in the (real or synthetic) sample
*I_s_*	A binary indicator of whether record *s* in the real sample matches a record in the synthetic sample
*R_s_*	A binary indicator of whether the adversary would learn something new if record *s* in the real sample matches a record in the synthetic sample
*k*	Number of quasi-identifiers
_λ_	Adjustment to account for errors in matching and a verification rate that is not perfect
*L*	The minimal percentage of sensitive variables that need to be similar between the real sample and synthetic sample to consider that an adversary has learned something new

### Adjusting for Incorrect Matches

In practice, 2 adjustments should be made to equation (6) to take into account the reality of matching when attempting to identify records [60]: data errors and the likelihood of verification. The overall probability can be expressed as:

*pr(a)pr(b*|*a)pr(c*|*a,b)*

*pr(a)* is the probability that there are no errors in the data, *pr(b*|*a)* is the probability of a match given that there are no errors in the data, and *pr(c*|*a,b)* is the probability that the match can be verified given that there are no errors in the data and that the records match.

Real data has errors in it, and therefore, the accuracy of the matching based on adversary knowledge will be reduced [53,61]. Known data error rates not specific to health data (eg, voter registration databases, surveys, and data from data brokers) can be relatively large [62-65]. For health data, the error rates have tended to be lower [66-70], with a weighted mean of 4.26%. Therefore, the probability of at least one variable having an error in it is given by 1–(1–0.0426)^k^, where *k* is the number of quasi-identifiers. If we assume that the adversary has perfect information and only the data will have an error in it, then the probability of no data errors is *pr(a)*=(1–0.0426)^k^.

A previous review of identification attempts found that when there is a suspected match between a record and a real individual, the suspected match could only be verified 23% of the time [71], *pr(c*|*a,b)*=0.23. This means that a large proportion of suspected matches turn out to be false positives when the adversary attempts to verify them. A good example from a published re-identification attack illustrating this is when the adversary was unable to contact the individuals to verify the matches in the time allotted for the study [11] (there are potentially multiple reasons for this, such as people moved, died, or their contact information was incorrect), which was 23%. It means that even though there is a suspected match, verifying it is not certain, and without verification, it would not be known whether the match was correct. In some of these studies, the verification ability is confounded with other factors, and therefore, there is uncertainty around this 23% value.

We can now adjust equation (6) with the λ parameter:

**λ=0.23×(1–0.0426)^k^ (8)**

However, equation (8) does not account for the uncertainty in the values obtained from the literature and assumes that verification rates and error rates are independent. Specifically, when there are data errors, they would make the ability to verify less likely, which makes these 2 effects correlated. We can model this correlation, as explained below.

The verification rate and data error rate can be represented as triangular distributions, which is a common way to model phenomena for risk assessment where the real distribution is not precisely known [72]. The means of the distributions are the values noted above, and the minimum and maximum values for each of the triangular distributions were taken from the literature (cited above).

We can also model the correlation between the 2 distributions to capture the dependency between (lack of) data errors and verification. This correlation was assumed to be medium, according to Cohen guidelines for the interpretation of effect sizes [73]. We can then sample from these 2 triangular distributions inducing a medium correlation [74]. The 2 sampled values can be entered into equation (8) instead of the mean values, and we get a new value, λ_s_, based on the sampled values. We draw from the correlated triangular distributions for every record in the real sample.

We can use the λ_s_ value directly in our model. However, to err on the conservative side and avoid this adjustment for data errors and verification over-attenuating the actual risk, we use instead the midpoint between λ_s_ and the maximum value of 1. We define

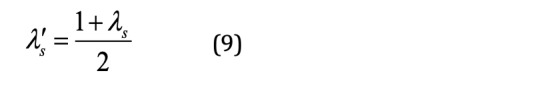



This more conservative adjustment can be entered into equation (6) as follows:

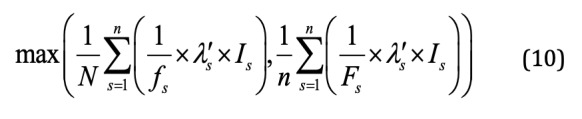



### Learning Something New

We now extend the risk model in equation (10) to determine if the adversary would learn something new from a match. We let *R_s_* be a binary indicator of whether the adversary could learn something new:

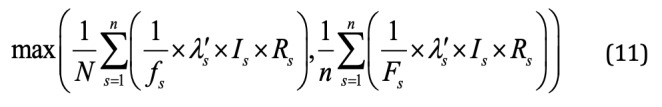



Because a real sample record can match multiple synthetic sample records, the *R_s_* is equal to 1 if any of the matches meets the “learning something new” threshold.

In practice, we compute *I_s_* first, and if that is 0, then there is no point in computing the remaining terms for that *s* record: we only consider those records that have a match between the real and synthetic samples since the “learning something new” test would not be applicable where there is no match.

Learning something new in the context of synthetic data can be expressed as a function of the sensitive variables. Also note that for our analysis, we assume that each sensitive variable is at the same level of granularity as in the real sample since that is the information that the adversary will have after a match.

The test of whether an adversary learns something new is defined in terms of 2 criteria: (1) Is the individual’s real information different from other individuals in the real sample (ie, to what extent is that individual an outlier in the real sample)? And (2) to what extent is the synthetic sample value similar to the real sample value? Both of these conditions would be tested for every sensitive variable.

Let us suppose that the sensitive variable we are looking at is the cost of a procedure. Consider the following scenarios: If the real information about an individual is very similar to other individuals (eg, the value is the same as the mean), then the information gain from an identification would be low (note that there is still some information gain, but it would be lower than the other scenarios). However, if the information about an individual is quite different, say the cost of the procedure is 3 times higher than the mean, then the information gain could be relatively high because that value is unusual. If the synthetic sample cost is quite similar to the real sample cost, then the information gain is still higher because the adversary would learn more accurate information. However, if the synthetic sample cost is quite different from the real sample cost, then very little would be learned by the adversary, or what will be learned will be incorrect, and therefore, the correct information gain would be low.

**Figure 2:**
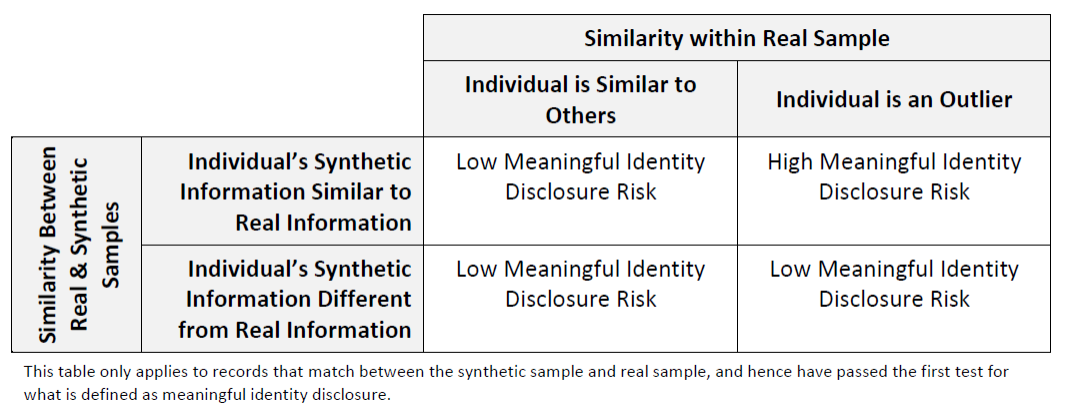
The relationship between a real observation to the rest of the data in the real sample and to the synthetic observation, which can be used to determine the likelihood of meaningful identity disclosure.

This set of scenarios is summarized in Figure 2. Only 1 quadrant (top right) would then represent a high and correct information gain, and the objective of our analysis is to determine whether a matched individual is in that quadrant for at least *L*% of its sensitive variables. A reasonable value of *L* would need to be specified for a particular analysis.

We propose a model to assess what the adversary would learn from each sensitive variable. If the adversary learns something new for at least *L%* of the sensitive variable, then we set *R*_2_=1; otherwise, it is 0.

### Nominal and Binary Sensitive Variables

We start off with nominal/binary sensitive variables and then extend the model to continuous variables. Let *X_s_* be the sensitive variable for real record *s* under consideration, and let *J* be the set of different values that *X_s_* can take in the real sample. Assume the matching record has value *X_s_=j* where *j∈J*, and that *p*_j_ is the proportion of records in the real sample that have the same *j* value.

We can then determine the distance that the *X_s_* value has from the rest of the real sample data as follows:

***d_j_=1–p_j_* (12)**

The distance is low if the value *j* is very common, and it is large if the value of *j* is very different than the rest of the real sample dataset.

Let the matching record on the sensitive variable in the synthetic record be denoted by *Y_t_=z*, where *z∈Z* and *Z* is the set of possible values that *Y*_t_ can take in the synthetic sample; in practice, *Z⊆J*. For any 2 records that match from the real sample and the synthetic sample, we compare their values. The measure of how similar the real value is to the rest of the distribution when it matches is therefore given by d_j_×[X_s_=Y_t_], where the square brackets are Iverson brackets.

How do we know if that value indicates that the adversary learns something new about the patient?

We set a conservative threshold; if the similarity is larger than 1 standard deviation, assuming that taking on value *j* follows a Bernoulli distribution, we then have the inequality for nominal and binary variables that must be met to declare that an adversary will learn something new from a matched sensitive variable.

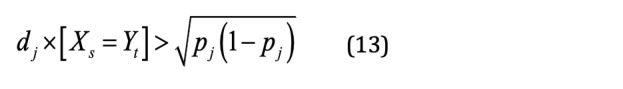



The inequality compares the weighted value with the standard deviation of the proportion *p_j_*.

### Continuous Sensitive Variables

Continuous sensitive variables should be discretized using univariate k-means clustering, with optimal cluster sizes chosen by the majority rule [75]. Again, let *X* be the sensitive variable under consideration, and *X_s_* be the value of that variable for the real record under consideration. We define the cluster's size in the real sample with the value of the sensitive variable that belongs to the matched real record under consideration as *C_s_*. For example, if the sensitive variable is the cost of a procedure and it is $150, and if that specific value is in a cluster of size 5, then *C_s_=5*. The proportion of all patients that are in this cluster compared to all patients in the real sample is given by *p_s_*.

In the same manner as for nominal and binary variables, the distance is defined as

***d_s_=p_s_* (14)**

Let *Y_t_* be the synthetic value on the continuous sensitive variable that matched with real record*s*. The weighted absolute difference expresses how much information the adversary has learned, d_s_×|*X_s_-Y_t_*|.

We need to determine if this value signifies learning too much. We compare this value to the median absolute deviation (MAD) over the *X* variable. The MAD is a robust measure of variation. We define the inequality:

**d_s_×|*X_s_–Y_t_*|<1.48×*MAD* (15)**

When this inequality is met, then the weighted difference between the real and synthetic values on the sensitive variable for a particular patient indicates that the adversary will indeed learn something new.

The 1.48 value makes the MAD equivalent to 1 standard deviation for Gaussian distributions. Of course, the multiplier for MAD can be adjusted since the choice of a single standard deviation equivalent was a subjective (albeit conservative) decision.

### Comprehensive Evaluation of Attacks

An adversary may not attempt to identify records on their original values but instead generalize the values in the synthetic sample and match those. The adversary may also attempt to identify records on a subset of the quasi-identifiers. Therefore, it is necessary to evaluate generalized values on the quasi-identifiers and subsets of quasi-identifiers during the matching process.

In Multimedia Appendix 1, we describe how we perform a comprehensive search for these attack modalities by considering all generalizations and all subsets, and then we take the highest risk across all combinations of generalization and quasi-identifier subsets as the overall meaningful identity disclosure risk of the dataset.

## Methods

We describe the methods used to apply this meaningful identity disclosure risk assessment model on 2 datasets.

### Datasets Evaluated

We apply the meaningful identity disclosure measurement methodology on 2 datasets. The first is the Washington State Inpatient Database (SID) for 2007. This is a dataset covering population hospital discharges for the year. The dataset has 206 variables and 644,902 observations. The second is the Canadian COVID-19 case dataset with 7 variables and 100,220 records gathered by Esri Canada [76].

We selected a 10% random sample from the full SID and synthesized it (64,490 patients). Then, meaningful identity disclosure of that subset was evaluated using the methodology described in this paper. The whole population dataset was used to compute the population parameters in equation (5) required for calculating the identity disclosure risk values according to equation (11). This ensured that there were no sources of estimation error that needed to be accounted for.

The COVID-19 dataset has 7 variables, with the date of reporting, health region, province, age group, gender, case status (active, recovered, deceased, and unknown), and type of exposure. A 20% sample was taken from the COVID-19 dataset (20,045 records), and the population was used to compute the meaningful identity disclosure risk similar to the Washington SID dataset.

### Quasi-identifiers

State inpatient databases have been attacked in the past, and therefore, we know the quasi-identifiers that have been useful to an adversary. One attack was performed on the Washington SID [11], and a subsequent one on the Maine and Vermont datasets [10]. The quasi-identifiers that were used in these attacks and that are included in the Washington SID are shown in Table 5.

**Table 5:** Quasi-identifiers included in the analysis of the Washington State Inpatient Database (SID) dataset.

Variable	Definition
AGE	patient's age in years at the time of admission
AGEDAY	age in days of a patient under 1 year of age
AGEMONTH	age in months for patients under 11 years of age
PSTCO2	patient's state/county federal information processing standard (FIPS) code
ZIP	patient's zip code
FEMALE	sex of the patient
AYEAR	hospital admission year
AMONTH	admission month
AWEEKEND	admission date was on a weekend

For the COVID-19 dataset, all of the variables, except exposure, would be considered quasi-identifiers since they would be knowable about an individual.

### Data Synthesis Method

For data synthesis, we used classification and regression trees [77], which have been proposed for sequential data synthesis [78] using a scheme similar to sequential imputation [79,80]. Trees are used quite extensively for the synthesis of health and social sciences data [34,81-88]. With these types of models, a variable is synthesized by using the values earlier in the sequence as predictors.

The specific method we used to generate synthetic data is called conditional trees [89], although other tree algorithms could also be used. A summary of the algorithm is provided in Textbox 1. When a fitted model is used to generate data, we sample from the predicted terminal node in the tree to get the synthetic values.

**Description of the sequential synthesis algorithm.**

Let us say that we have 5 variables, A, B, C, D, and E. The generation is performed sequentially, and therefore, we need to have a sequence. Various criteria can be used to choose a sequence. For our example, we define the sequence as A→E→C→B→D.

Let the prime notation indicate that the variable is synthesized. For example, A’ means that this is the synthesized version of A. The following are the steps for sequential generation:

- Sample from the A distribution to get A’
- Build a model F1: E ∼ A
- Synthesize E as E’ = F1(A’)
- Build a model F2: C ∼ A + E
- Synthesize C as C’ = F2(A’, E’)
- Build a model F3: B ∼ A + E + C
- Synthesize B as B’ = F3(A’, E’, C’)
- Build a model F4: D ∼ A + E + C + B
- Synthesize D as D’ = F4(A’, E’, C’, B’)

The process can be thought of as having 2 steps, fitting and synthesis. Initially, we are fitting a series of models (F1, F2, F3, F4). These models make up the generator. Then these models can be used to synthesize data according to the scheme illustrated above.

### Risk Assessment Parameters

As well as computing the meaningful identity disclosure risk for the synthetic sample, we computed the meaningful identity disclosure risk for the real sample itself. With the latter, we let the real sample play the role of the synthetic sample, which means we are comparing the real sample against itself. This should set a baseline to compare the risk values on the synthetic data and allows us to assess the reduction in meaningful identity disclosure risk due to data synthesis. Note that both of the datasets we used in this empirical study were already de-identified to some extent.

For the computation of meaningful identity disclosure risk, we used an acceptable risk threshold value of 0.09 to be consistent with values proposed by large data custodians and have been suggested by the European Medicines Agency and Health Canada for the public release of clinical trial data (Multimedia Appendix 1). We also set *L*=5%.

### Ethics

This study was approved by the CHEO Research Institute Research Ethics Board, protocol numbers 20/31X and 20/73X.

## Results

The meaningful identity disclosure risk assessment results according to equation (11) for the Washington hospital discharge data are shown in Table 6. We can see that the overall meaningful identity disclosure risk for the synthetic data is significantly lower than the threshold of 0.09. We compare this to the real data, where the overall reduction in risk due to synthesis is approximately 5 times. The synthetic data is 4.5 times below the threshold.

The risk result on the real dataset is consistent with the empirical attack results [11]: An attempt to match 81 individuals resulted in verified, correct matches of 8 individuals, which is a risk level of 0.099 and is more or less the same as the value that was calculated using the current methodology. The real data risk was higher than the threshold, and therefore, by this standard, the original dataset would be considered to have an unacceptably high risk of identifying individuals.

The results for the synthetic Canadian COVID-19 case data are also below the threshold by about 10 times, and 4 times below risk values for the real data, although the original data has a risk value that is also below the threshold.

However, it is clear that the synthetic datasets demonstrate a significant reduction in meaningful identity disclosure risk compared to the original real dataset.

**Table 6:** Overall meaningful identity disclosure risk results. (The italicized values are the maximum risk values.)

Parameter	Synthetic data risk		Real data risk	
	Population-to-sample risk	Sample-to-population risk	Population-to-sample risk	Sample-to-population risk
Washington State Inpatient Database	0.00056	*0.0197*	0.016	*0.098*
Canadian COVID-19 cases	0.0043	*0.0086*	0.012	*0.034*

## Discussion

### Summary

The objective of this study was to develop and empirically test a methodology for the evaluation of identity disclosure risks for fully synthetic health data. This methodology builds on previous work on attribution risk for synthetic data to provide a comprehensive risk evaluation. It was then applied to a synthetic version of the Washington hospital discharge database and the Canadian COVID-19 cases dataset.

We found that the meaningful identity disclosure risk was below the commonly used risk threshold of 0.09 between 4.5 times and 10 times. Note that this reduced risk level was achieved without implementing any security and privacy controls on the dataset, suggesting that the synthetic variant can be shared with limited controls in place. The synthetic data also had a lower risk than the original data by between 4 and 5 times.

These results are encouraging in that they provide strong empirical evidence to claims in the literature that the identity disclosure risks from fully synthetic data are low. Further tests and case studies are needed to add more weight to these findings and determine if they are generalizable to other types of datasets.

### Contributions of this Research

This work extends, in important ways, previous privacy models for fully synthetic data. Let *R’_s_* be an arbitrary indicator of whether an adversary learns something new about a real sample record *s*. An earlier privacy risk model [42,47] focused on attribution risk was defined as:

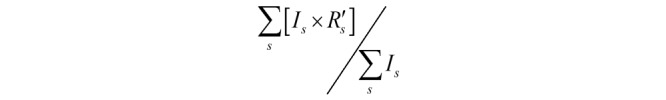



This is similar to our definition of learning something new conditional on identity disclosure. Our model extends this work by also considering the likelihood of matching the real sample record to the population using both directions of attack, including a comprehensive search for possible matches between the real sample and synthetic sample. We also consider data errors and verification probabilities in our model, and our implementation of *R’_s_* allows for uncertainty in the matching beyond equality tests.

Some previous data synthesis studies examined another type of disclosure: membership disclosure [28,48]. The assessment of meaningful identity disclosure, as described in this paper, does not preclude the evaluation of membership disclosure when generating synthetic data, and in fact, both approaches can be considered as complementary ways to examine privacy risks in synthetic data.

Privacy risk measures that assume that an adversary has white-box or black-box access to the generative model [29] are not applicable to our scenario, as our assumption has been that only the synthetic data is shared and the original data custodian retains the generative model.

### Applications in Practice

Meaningful identity disclosure evaluations should be performed on a regular basis on synthetic data to ensure that the generative models do not overfit. This can complement membership disclosure assessments, providing 2 ways of performing a broad evaluation of privacy risks in synthetic data.

With our model, it is also possible to include meaningful identity disclosure risk as part of the loss function in generative models to simultaneously optimize on identity disclosure risk as well as data utility, and to manage overfitting during synthesis since a signal of overfitting would be a high meaningful identity disclosure risk.

### Limitations

The overall risk assessment model is agnostic to the synthesis approach that is used; however, our empirical results are limited to using a sequential decision tree method for data synthesis. While this is a commonly used approach for health and social science data, different approaches may yield different risk values when evaluated using the methodology described here.

We also made the worst-case assumption that the adversary knowledge is perfect and is not subject to data errors. This is a conservative assumption but was made because we do not have data or evidence on adversary background knowledge errors.

Future work should extend this model to longitudinal datasets, as the current risk model is limited to cross-sectional data.

## Appendix

Multimedia Appendix 1 Details of calculating and interpreting identity disclosure risk values.

## Abbreviations

AIML: artificial intelligence and machine learning
MAD: median absolute deviation
SID: State Inpatient Database
