# Supplementary material for: Evaluating Identity Disclosure Risk in Fully Synthetic Health Data: Model Development and Validation
Source: J Med Internet Res. 2020 Nov 16;22(11):e23139. doi: 10.2196/23139 (PMC7704280; doi:10.2196/23139)
Supplement: Multimedia Appendix 1 [file jmir_v22i11e23139_app1.pdf]

Appendix A: Evaluating Possible Attacks

A.1 Matching with Generalization

Generalizations on the quasi-identifiers can be expressed in terms of the example hierarchies as illustrated for the three quasi-identifiers in Figure 1.

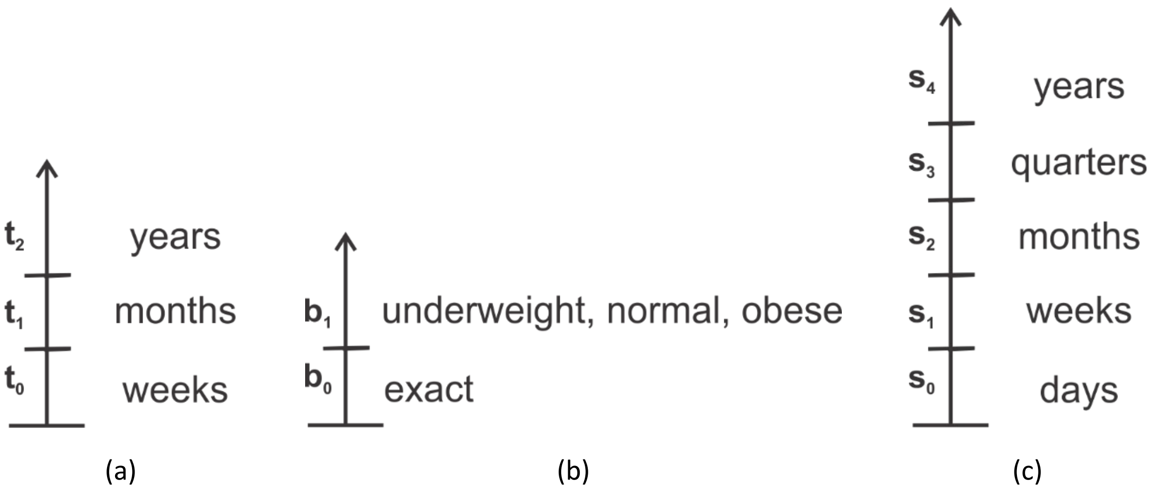

Figure 1: Examples of three generalization hierarchies for (a) time on the study for say a clinical trial, (b) BMI, and (c) survival. As one moves up the hierarchy the level of granularity decreases, and this level is indicated by the notation on the left.

The more generalizations that are applied to the quasi-identifiers in the synthetic and real samples, the greater the chance of a match between the synthetic sample and the real sample. For example, if we try to match the synthetic sample with the real sample on the exact BMI then we may not match any patients, but if we generalize that to the b_1 categories in panel (b) in Figure 1, then the match rate does increase. At the same time, as the data are generalized, the risk of identification of the real data will likely decrease. Therefore, as one moves vertically through the lattice, the value in equation (11) in the main body of the paper will not necessarily change monotonically in either direction.

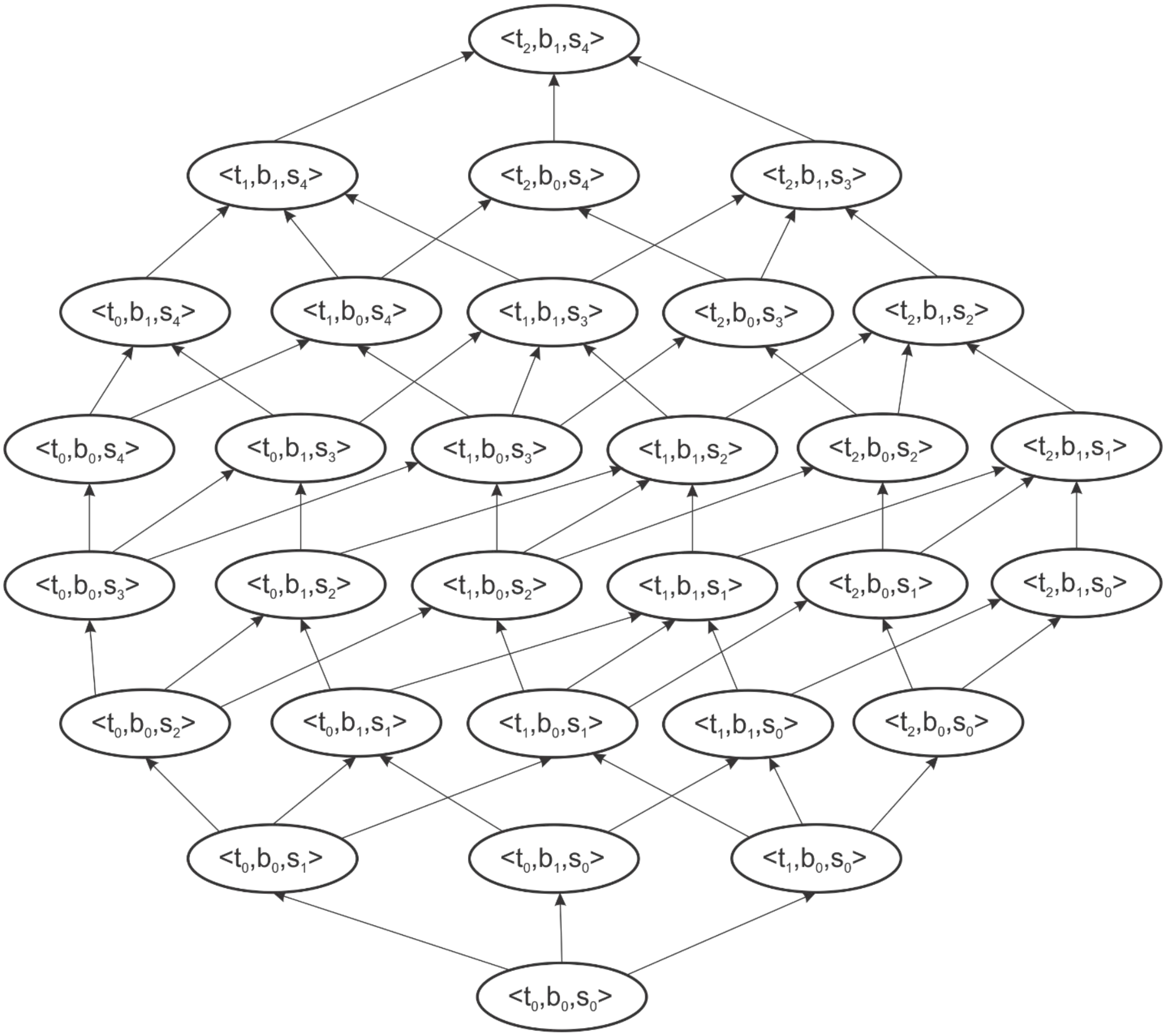

Figure 2: Generalization lattice for the three generalization hierarchies.

We can represent all possible generalizations on the quasi-identifiers as a *generalization lattice* (see Figure 2). In a generalization lattice the least generalized version of the quasi-identifiers is at the bottom (i.e., the highest granularity which is the original data), and the top of the lattice is the most generalized version of the quasi-identifiers. Each node represents a further generalization of a single quasi-identifier compared to the nodes below it in the lattice. The lattice represents all possible generalizations that an adversary can attempt on the synthetic data to identify it.

As we move up the lattice the likelihood of a match between the real sample and synthetic sample will by definition stay the same or *increase*. Also, as we move up the lattice, the risk of identification (the probability of matching a real sample record with a real person in the population or vice versa) will by definition stay the same or *decrease*.

The objective of navigating the lattice is to find the node that has the highest risk of meaningful identity disclosure, and that node represents the risk for the synthetic dataset. This means we assume that the

adversary will be able to identify the configuration of generalization that would maximize identity disclosure risk, and focus on that. This is somewhat conservative assumption.

A.2 Matching with Variable Subsets

If the number of quasi-identifiers is denoted by k, an adversary may also try to match on fewer than k variables. By definition, the fewer the number of variables the more likely it is to match synthetic records with real records. At the same time, the identification risk will decrease the fewer quasi-identifiers are considered.

The different combinations of quasi-identifiers can be represented as a *subsets lattice* as illustrated in Figure 3. At the bottom of the lattice are all of the quasi-identifiers, and as we go further up the lattice the number of variables decreases. We need to evaluate the risk in equation (11) in the main body of the paper or every node in the subsets lattice as well.

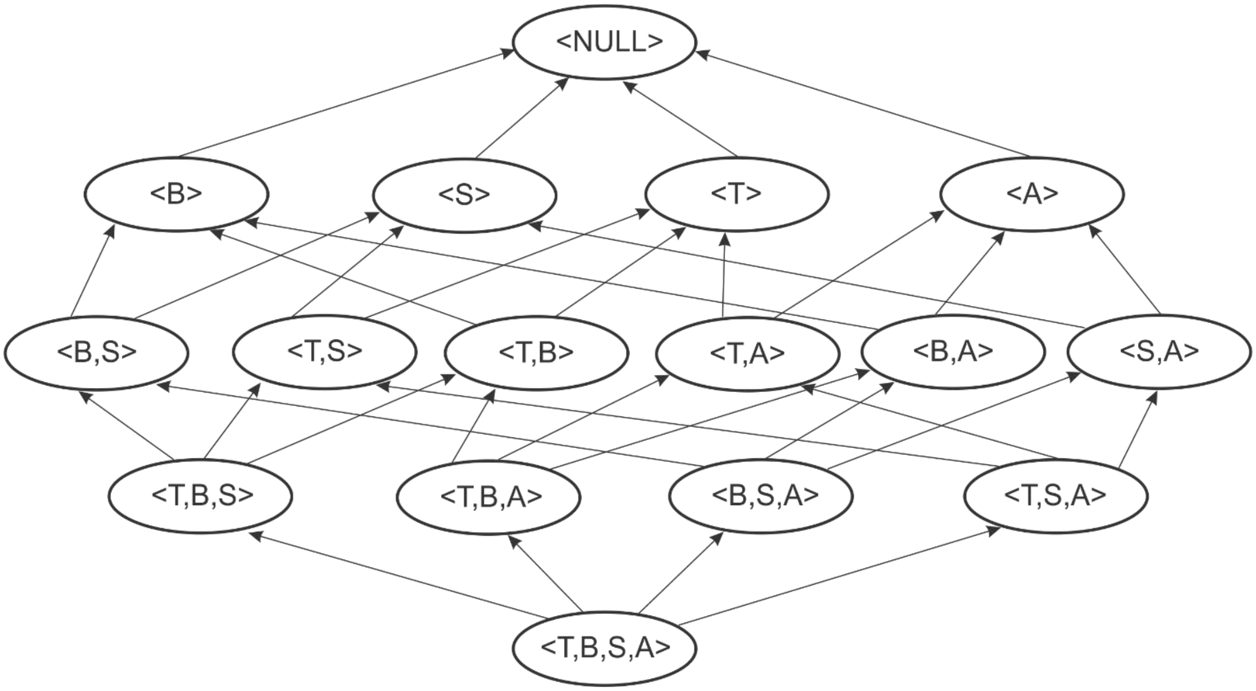

Figure 3: An example of a quasi-identifier subsets lattice with four quasi-identifiers: time on the study (T), BMI (B), survival (S), and adverse event (A).

A.3 Reducing Complexity

If we evaluate every node in each lattice then we have to compute risk for the following number of nodes:

$$\left(\prod_{i=1}^{k} Q_i\right)\times\left(1+\sum_{i=1}^{k}\binom{k}{i}\right) \quad (1)$$

where Q_i is the number of generalization levels in quasi-identifier i. Therefore, for example, the three quasi-identifiers with the hierarchies in Figure 1 would give us 240 nodes to compute the meaningful identity disclosure risk for to find the node with the highest risk value. However, the datasets that we are computing on become smaller as we move down the lattice, as explained below.

Initially, the R_s value can be computed for all records, and the real sample records with $R_s = 0$ eliminated from further consideration.

We can start our search from the top node in the generalization lattice, and as we move down, remove records from the real sample that were not matched. This means that the dataset size that is being processed to perform the calculation in equation (2) will gradually decrease as we move from top to bottom, speeding up the computations.

To take advantage of that pattern to reduce the amount of computation, every node need only consider the patients who have matched in nodes higher up in the lattice hierarchy along the defined generalization paths, as is illustrated in Figure 4. In this case, the intersection of real sample patients that matched (i.e., $I_s = 1$) in nodes <t_1,b_0,s_4>, <t_1,b_1,s_3> and <t_2,b_0,s_3> will be used to perform the computations in <t_1,b_0,s_3> (see Figure 4).

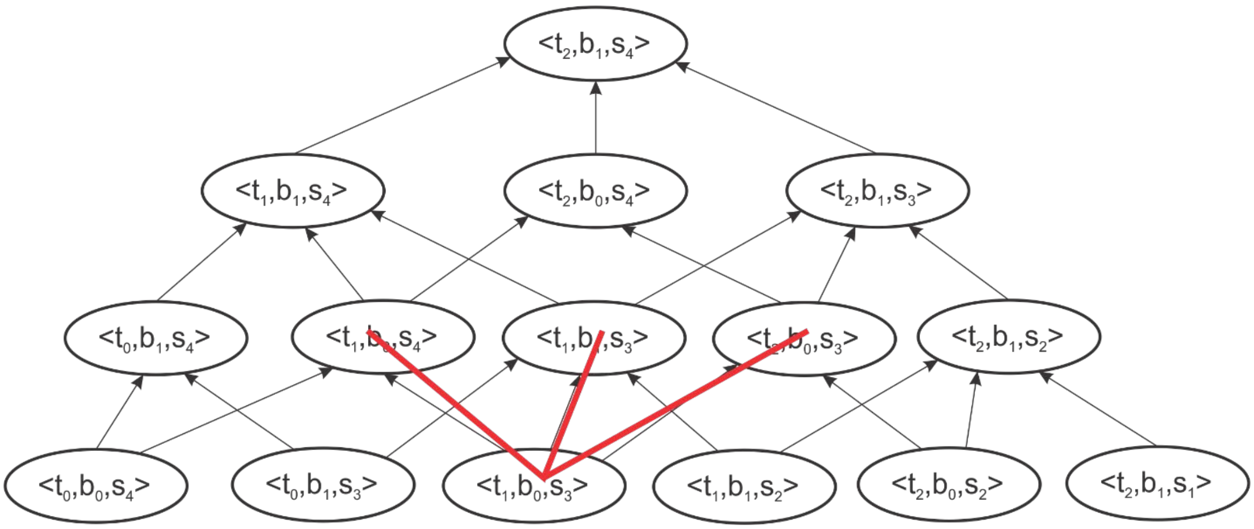

Figure 4: Performing analysis on the subset of patients defined by the intersection of patients from the nodes above in the generalization path.

In practice, the lattice navigation would start with the variable subsets lattice, and for every node there perform the computations on the generalization lattice for that subset of quasi-identifiers. Also, computations on the subsets lattice should start from the top moving down.

For every node in the generalization lattice, unmatched record elimination can be performed on the nodes above it in the subsets lattice. For example, node <t_0,b_0,s_2> would eliminate unmatched records in node <t_0,b_0>,<t_0,s_2>,<b_0,s_2>,<t_0>,<b_0>,<s_2> and those above them in their respective generalization lattice. This can reduce computation load overall.

If the highest risk node meets the following inequality, then the synthetic dataset is considered to have acceptably low meaningful identify disclosure risk:

$$\max\left(\frac{1}{N}\sum_{s=1}^{n}\left(\frac{1}{f_s}\times\lambda_s'\times I_s\times R_s\right),\frac{1}{n}\sum_{s=1}^{n}\left(\frac{1}{F_s}\times\lambda_s'\times I_s\times R_s\right)\right)\leq\tau \quad (2)$$

where τ is a threshold reflecting acceptable risk. We will discuss further below how to set a threshold.

Appendix B: Defining Acceptable Risk

For the purposes of our risk assessment model, the threshold τ is an important parameter because it is used to determine if the dataset is high risk or not. Below we provide some precedent-based guidance for choosing a value.

The general idea of there being a threshold for what is deemed acceptable identification risk is reflected qualitatively in various statutes. For example, under the HIPAA Privacy Rule, the risk needs to be "very small" that the information could be used, alone or in combination with other reasonably available information, by an anticipated recipient, to identify an individual [1]. Recent guidance from the Ontario Information and Privacy Commissioner's Office indicates that the risk of identification of individuals in data should be determined to be "very low" or "very small" prior to the data being released [2].

In Canada and the European Union, the reasonableness standard is widely used to judge whether or not information is identifiable [3]–[6]. For instance, in the Federal Court of Canada case of *Gordon v. Canada (Health)*,[1] Gibson, J. adopted the "serious possibility" test proposed by the Federal Privacy Commissioner to determine if information constitutes personal information as defined in the *Privacy Act*[2] (i.e. whether it is information about an identifiable individual).

Recital 26 of the European General Data Protection Regulation states, "To determine whether a natural person is identifiable, account should be taken of all the means reasonably likely to be used, such as singling out, either by the controller or by another person to identify the natural person directly or indirectly." [5] The EU Article 29 Working Party Opinion on Anonymization Techniques indicates that anonymized data must meet three specific criteria (must not allow for singling out, linkability or inference) or a re-identification risk analysis must be performed to demonstrate that the risk is "acceptably small" [7].

A key question then is how does one define subjective terms such as "reasonable", "reasonably likely", "serious possibility", "very low", "very small", or "acceptably small" risk ? And do so in a manner that can be practically translated into a probability value to set an identifiability threshold so that precise measurements can be applied ? Subjective translation of these terms to quantitative values proves to be difficult, with a large variation in how they are interpreted, according to recent surveys [8].

However, there are precedents for setting thresholds in the area of statistical disclosure control. The field of statistical disclosure control has historically concerned itself with the potential disclosure from small "cell sizes" when data is aggregated. That is, a cell in a table in which data is aggregated is considered sensitive if the number of individuals represented in the cell is below a threshold. This threshold rule is often called a minimum cell size. We therefore review existing precedents for setting "minimum cell sizes" since that is the dominant approach that has been applied thus far for deciding acceptable identifiability risk.

The threshold probability of identification is one divided by the minimum cell size. For example, if the minimum cell size is 5, then the threshold probability of re-identification is 1/5 or 0.2.

When governments and other organizations release statistical data, cell size thresholds are often used to ensure that the risk of identification remains at an acceptably low level. Historically, data custodians have used the "cell size of five" rule as a threshold for deciding whether to de-identify data [9]–[23]. This

1 2008 FC 258 (CanLII) 2008-02-27, at: https://www.canlii.org/en/ca/fct/doc/2008/2008fc258/2008fc258.html?searchUrlHash=AAAAAQAkR29yZG9uICAiUHJpdmFjeSBBY3QiIGFjY2VzcyBmZWRlcmFsAAAAAAE&resultIndex=2.

2 R.S.C. 1985, c. P-21.

rule was originally applied to count data in tables. Count data, however, can be easily converted to individual-level data—therefore these two representations are in effect the same thing. A minimum "cell size of five" rule would translate into a maximum probability of identifying a single record of 0.2. Some custodians use a cell size of 3 [24]–[28], which is equivalent to a probability of identifying a single record of 0.33. For the release of data a cell size of 11 has been used in the US [29]–[33], and a cell size of 20 for public Canadian and US patient data [34], [35], [36]. Cell sizes from 5 to 30 have been used across the US to protect student's personally identifying information, with the cell size of 10 being used most commonly (by 39 states) [37]. Other cell sizes such as 4 [38], 6 [39]–[43], 10 [36], [44]–[46], and 16 [47] have been used in different scenarios within varying countries.

The European Medicines Agency (EMA) has recently established a policy on the publication of clinical data for medicinal products [48] which requires applicants/sponsors to openly share clinical trial data. The guidelines accompanying the policy recommend a maximum risk threshold of 0.09, which is equivalent to a minimal cell size of 11. Health Canada implemented the same threshold for the sharing of clinical trial data [49].

In commentary about the de-identification standard in the HIPAA Privacy Rule, the US Department of Health and Human Services notes in the Federal Register that the identity disclosure risk is largely from unique records [18], [50]. Unique records are those that make up a cell count of 1. It is clear that they considered records that are not unique to have an acceptably low risk of being identified. This translates to a minimal cell size of 2. Although cell sizes less than three are not recommended in the disclosure control literature [51].

One would assume that there is a higher minimal cell size for more sensitive information. However, in such cases the thresholds used have not been so consistent. For example, a minimal count of 3 has been recommended for HIV/AIDS data [27], [52], a cell size of 5 for abortion data [13], and a cell size of 6 for at-risk children [53]. Recognizing that the type of data can differ, NHS Scotland has set a minimum cell size of 3 for non-sensitive data and a minimum cell size of 5 or 10 for sensitive information [54].

Therefore, two thresholds that can be used and that are based on strong precedents. The first is 0.2 since it is the most commonly used in practice. Another one that is more specific to health data is 0.09 since that has been explicitly proposed by two health regulators. The latter value is more conservative and provides a buffer for increasing adversary knowledge over time, and therefore it is the value that we recommend.

References

[1] US Congress, "The Health Insurance Portability and Accountability Act of 1996; 42 U.S. Code § 1320d - Definitions." 1996, [Online]. Available: http://bit.ly/2zmpuRs.

[2] Information and Privacy Commissioner of Ontario, "De-identification Guidelines for Structured Data," Jun. 2016. [Online]. Available: http://bit.ly/1PkrnMF.

[3] Government of Ontario, *Personal Health Information Protection Act*. 2004.

[4] Province of Alberta, *Health Information Act*. 2016, p. Chapter H-5.

[5] European Parliament and the Council of the European Union, *REGULATION (EU) NO 2016/679 OF THE EUROPEAN PARLIAMENT AND OF THE COUNCIL OF APRIL 27, 2016, on the protection of individuals with regard to the processing of personal data and on the free movement of such data, and repealing Directive 95/46/EC (General Data Protection Regulation).*, vol. NO 2016/679. 2016.

[6] Province of New Brunswick, *Personal Health Information Privacy and Access Act, SNB 2009, c P-7.05*.

[7] Article 29 Data Protection Working Party, "Opinion 05/2014 on Anonymization Techniques," Apr. 2014.

[8] A. Mauboussin and M. J. Mauboussin, "If You Say Something Is 'Likely,' How Likely Do People Think It Is?," *Harvard Business Review*, Jul. 03, 2018.

[9] "Security and confidentiality policies and procedures," Health Quality Council, 2004.

[10] "Privacy code," Manitoba Center for Health Policy, 2002.

[11] "Privacy code," Health Quality Council, 2004.

[12] Subcommittee on Disclosure Limitation Methodology, "Working paper 22: Report on statistical disclosure control," Office of Management and Budget, 1994.

[13] Statistics Canada, "Therapeutic Abortion Survey." 2007, [Online]. Available: http://bit.ly/2AnRaDN.

[14] Office of the Information and Privacy Commissioner of British Columbia, "Order No. 261-1998," 1998.

[15] Office of the Information and Privacy Commissioner of Ontario, "Order P-644." 1994, [Online]. Available: http://bit.ly/2zmoQDC.

[16] L. Alexander and T. Jabine, "Access to social security microdata files for research and statistical purposes," *Social Security Bulletin*, vol. 41, no. 8, pp. 3–17, 1978.

[17] Ministry of Health and Long Term care (Ontario), "Corporate Policy 3-1-21," 1984.

[18] Department of Health and Human Services, "Standards for privacy of individually identifiable health information," 2000. [Online]. Available: http://bit.ly/2hi7EbF.

[19] "Guidelines for the Public Release of Public Health Data," New Hampshire Department of Health and Human Services, Sep. 2008. [Online]. Available: http://www.dhhs.nh.gov/dphs/hsdm/documents/publichealthdata.pdf.

[20] Research Data Center, "Disclosure Manual - Preventing Disclosure: Rules for Researchers," National Center for Health Statistics, Mar. 2012. [Online]. Available: http://bit.ly/2zlXbke.

[21] Public Health England, "PHE HIV & STI Data Sharing Policy," May 2014. [Online]. Available: http://bit.ly/2hgosjv.

[22] "Data Confidentiality / IRB Policy." Institute for Community Health, Accessed: Apr. 13, 2015. [Online]. Available: http://bit.ly/2Ao1T0M.

[23] "Internal Guidelines for Working with Small Cell Sizes," Office of AIDS - California Department of Public Health, Sep. 2014. Accessed: Feb. 29, 2016. [Online]. Available: http://bit.ly/2zkBoct.

[24] A. de Waal and L. Willenborg, "A View on Statistical Disclosure Control for Microdata," *Survey Methodology*, vol. 22, no. 1, pp. 95–103, 1996.

[25] G. Duncan, T. Jabine, and S. de Wolf, *Private Lives and Public Policies: Confidentiality and Accessibility of Government Statistics*. National Academies Press, 1993.

[26] Office of the Privacy Commissioner of Quebec (CAI), "Chenard v. Ministere de l'agriculture, des pecheries et de l'alimentation (141)," 1997.

[27] Centers for Disease Control and Prevention, "Integrated Guidelines for Developing Epidemiologic Profiles: HIV Prevention and Ryan White CARE Act Community Planning," 2004.

[28] "Disclosure control guidance for birth and death statistics," Office for National Statistics, Jan. 2014.

[29] CMS, "2008 Basic Stand Alone Medicare Claims Public Use Files." http://go.cms.gov/2itDh2o.

[30] E. Erdem and S. I. Prada, "Creation of public use files: lessons learned from the comparative effectiveness research public use files data pilot project," Sep. 13, 2011. http://bit.ly/2xZKfyb (accessed Nov. 09, 2012).

[31] "Instructions for Completing the Limited Data Set ATA use Agreement (DUA) (CMS-R-0235L)." Department of Health & Human Services, [Online]. Available: http://go.cms.gov/2yJ1KX4.

[32] California Department of Health Care Services, "Public Reporting Guidelines." https://www.dhcs.ca.gov/dataandstats/Pages/PublicReportingGuidelines.aspx (accessed May 23, 2020).

[33] State of Vermont Agency of Education, "Data Governance." https://education.vermont.gov/data-and-reporting/data-governance (accessed May 23, 2020).

[34] K. El Emam, D. Paton, F. Dankar, and G. Koru, "De-identifying a Public Use Microdata File from the Canadian National Discharge Abstract Database," *BMC Medical Informatics and Decision Making*, vol. 11, no. 53, 2011.

[35] K. El Emam *et al.*, "De-identification Methods for Open Health Data: The Case of the Heritage Health Prize Claims Dataset," *Journal of Medical Internet Research*, vol. 14, no. 1, p. e33, Feb. 2012, doi: 10.2196/jmir.2001.

[36] Data Suppression Decision Rules Work Group, "Report of Guidelines for Data Result Suppression," Utah Department of Health, Oct. 2009. [Online]. Available: http://bit.ly/2yBVGBi.

[37] National Center for Education Statistics, "SLDS Technical Brief 3: Statistical Methods for Protecting Personally Identifiable Information in Aggregate Reporting," Institute of Education Sciences, Dec. 2010. [Online]. Available: http://bit.ly/1UsB2F9.

[38] "Policy for Disclosure of Reportable Disease Information," Iowa Department of Public Health, Nov. 2005. [Online]. Available: http://bit.ly/2hMvpWr.

[39] Health & Social Care Information Centre, "The HES Protocol," Jun. 2009. [Online]. Available: http://bit.ly/2h99JmJ.

[40] "2013 Census confidentiality rules and how they are applied," Statistics New Zealand, 2013. [Online]. Available: http://bit.ly/2lWDaOa.

[41] "Department of Public Health Confidentiality Procedures," Massachusetts Executive Office of Health and Human Services, Oct. 2012. [Online]. Available: http://bit.ly/2j8W2IR.

[42] "MIYHS Data Release Policy." Office of Substance Abuse and Mental Health Services, the Maine Center for Disease Control and Prevention, and the Department of Education, Feb. 2010, [Online]. Available: http://bit.ly/2hasqGw.

[43] "Data Use & Disclosure Standard," Cancer Care Ontario, Sep. 2011. Accessed: Mar. 11, 2016. [Online]. Available: http://bit.ly/2AlrWWp.

[44] C. J. Harrell, K. A. Herget, C. L. Bateman, and R. Dibble, “Cancer in Utah,” Utah Cancer Registry, 2014. Accessed: Feb. 29, 2016. [Online]. Available: http://bit.ly/2yA3J1n.

[45] “Data Methodology Standards for Public Health Practice,” Ohio Department of Health. Accessed: Feb. 26, 2016. [Online]. Available: http://bit.ly/2zjzQlz.

[46] “Guidelines for Working with Small Numbers,” Washington State Department of Health, Oct. 2012. Accessed: Feb. 23, 2016. [Online]. Available: http://www.doh.wa.gov/portals/1/documents/5500/smallnumbers.pdf.

[47] “Technical Notes: Statistical Methods: Suppression of Rates and Counts.” United States Cancer Statistics, Sep. 2014, [Online]. Available: http://www.cdc.gov/cancer/npcr/uscs/technical_notes/stat_methods/suppression.htm.

[48] European Medicines Agency, “European Medicines Agency policy on publication of data for medicinal products for human use: Policy 0070.” Oct. 02, 2014, [Online]. Available: http://www.ema.europa.eu/docs/en_GB/document_library/Other/2014/10/WC500174796.pdf.

[49] Health Canada, “Guidance document on Public Release of Clinical Information,” Apr. 01, 2019. https://www.canada.ca/en/health-canada/services/drug-health-product-review-approval/profile-public-release-clinical-information-guidance.html.

[50] Department of Health and Human Services, “Standards for privacy of individually identifiable health information. Federal Register,” 2000. [Online]. Available: http://aspe.hhs.gov/admnsimp/final/PvcFR05.txt.

[51] L. Willenborg and T. de Waal, *Elements of Statistical Disclosure Control*. New York: Springer-Verlag, 2001.

[52] “Arkansas HIV/AIDS Data Release Policy,” Arkansas Department of Health, Mar. 2010. [Online]. Available: http://www.healthy.arkansas.gov/programsServices/healthStatistics/Documents/STDSurveillance/Datadeissemination.pdf.

[53] United Kingdom, “Statistical Policy Statement on Confidentiality,” Apr. 2013. [Online]. Available: https://www.gov.uk/government/uploads/system/uploads/attachment_data/file/190768/Confidentiality_Policy_v4.pdf.

[54] “ISD Statistical Disclosure Control Protocol,” NHS National Services Scotland, Jun. 2010. Accessed: Mar. 16, 2016. [Online]. Available: http://www.isdscotland.org/About-ISD/About-Our-Statistics/isd-statistical-disclosure-protocol.pdf.
